# Qualitative and quantitative spermatic cord abnormalities at CT predict symptomatic scrotal pathology

**DOI:** 10.1007/s00261-024-04251-6

**Published:** 2024-03-22

**Authors:** Ryan T. Whitesell, John F. Brunner, Heather R. Collins, Douglas H. Sheafor

**Affiliations:** 1 Midwest Radiology, 2355 Highway 36 West, Roseville, MN USA; 2 Department of Radiology, Medical University of South Carolina, Charleston, SC USA

**Keywords:** Spermatic cord, Scrotum, Multidetector computed tomography, Ultrasound

## Abstract

**Purpose:**

To evaluate quantitative and qualitative spermatic cord CT abnormalities and presence of unilateral or bilateral symptomatic scrotal pathology (SSP) at ultrasound.

**Methods:**

This retrospective study included 122 male patients (mean age 47.8 years) undergoing scrotal ultrasound within 24 h of contrast-enhanced CT (n = 85), non-contrast CT (NECT, n = 32) or CT-Urogram (n = 5). CECT quantitative analysis assessed differential cord enhancement using maximum Hounsfield unit measurements. Three fellowship trained body radiologists independently assessed qualitative cord abnormalities for both CECT and NECT. Qualitative and quantitative findings were compared with the presence of SSP. Reader performance, interobserver agreement and reader confidence were assessed for NECT and CECT. Quantitative cutoff points were identified which maximized accuracy, specificity, negative predictive value, and other measures.

**Results:**

SSP was present in 36/122 patients (29.5%). Positive cases were unilateral in 30 (83.3%) and bilateral in 6 (16.6%). At quantitative assessment, 25% differential cord enhancement had the highest diagnostic accuracy (88.9%), with 90.5% positive predictive value, 88.4% negative predictive value, 96.8% specificity, and 70.4% sensitivity. At qualitative evaluation, CECT reader performance was excellent (aggregate AUC = 0.86; *P* < .001); NECT was poorly discriminatory, although remained significant (aggregate AUC = 0.67; *P* = .002). Readers had significantly higher confidence levels with CECT (*P* < .001). Qualitative inter-observer agreement was high for both CECT and NECT (ICC = 0.981 and 0.963, respectively).

**Conclusion:**

Simple quantitative assessment of differential cord enhancement is highly accurate and specific for SSP at CECT. Qualitative abnormalities at CECT and NECT are also both predictors of SSP, however, CECT significantly out-performs non-contrast exams.

**Graphical abstract:**

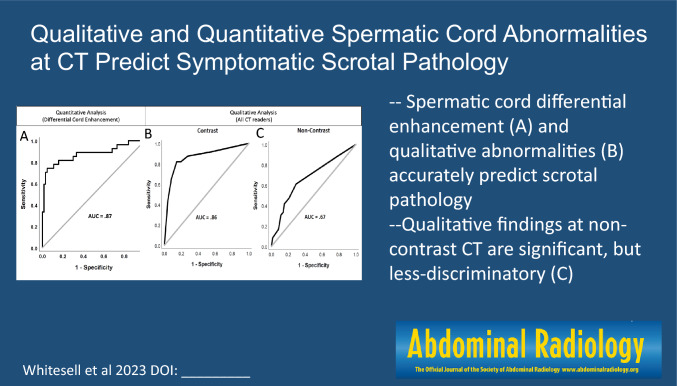

## Introduction

In male patients with scrotal pain or swelling, the differential diagnosis is broad. In the emergency department, patients with typical presentations are usually evaluated with scrotal ultrasound, which is readily available, is relatively inexpensive, and produces no ionizing radiation [1, 2]. In these cases, ultrasound is both sensitive and specific in the detection of the most common scrotal pathologies, such as epididymitis, orchitis, testicular torsion, and testicular neoplasm [3–7]. Unfortunately, scrotal pathology does not always present with a clear clinical picture, sometimes due to referred pain into the flank, pelvis, or hip; conversely, intra-abdominal and pelvic pathologies may result in scrotal or groin pain [8, 9]. Additionally, in busy Emergency Departments, imaging may be ordered after only a rapid triage assessment, which could result in larger numbers in patients with scrotal pathology being screened with initial computed tomography (CT), bypassing sonographic evaluation [10, 11]. While routine abdominopelvic CT includes portions of the spermatic cord, the entire scrotum is not typically imaged, which could limit detection of symptomatic scrotal pathology (SSP). In our experience, it is also not uncommon to discover CT cord abnormalities in retrospect, following sonographic diagnosis of SSP. This may be due in part to the location of the spermatic cords at “the corner of the film”, or potentially due to differential cord enhancement being discounted as a varicocele.

Two retrospective studies have described enlargement and/or hyper-enhancement of the ipsilateral spermatic cord as important secondary findings which may be seen in the setting of scrotal pathology at contrast-enhanced CT [12, 13]. Both studies showed a correlation between spermatic cord findings at contrast-enhanced CT (CECT) and scrotal pathology in small sample sizes, however, bilateral scrotal abnormalities were excluded. The first relied on detailed vascular measurements in an outpatient patient population primarily with varicoceles [12]. Qualitative techniques used in the subsequent study may be less reliable in centers with variable CT protocols and did not establish thresholds for use with prospective reads [13]. Neither study included findings at non-contrast CT (NECT). This could be significant as NECT is frequently performed in patients with flank and groin pain for suspected urolithiasis—symptoms which may overlap with acute scrotal pathology. Therefore, the purpose of this study was to validate a new spermatic cord quantitative assessment technique in a larger patient cohort, and to correlate CECT and NECT abnormalities with the presence of SSP.

## Materials and methods

This retrospective study was approved by the Institutional Review Board of Health Partners Institute; informed consent for review of medical records and imaging was waived. All study activities were performed in compliance with HIPAA.

### Study population and patient selection

**Fig. 1:**
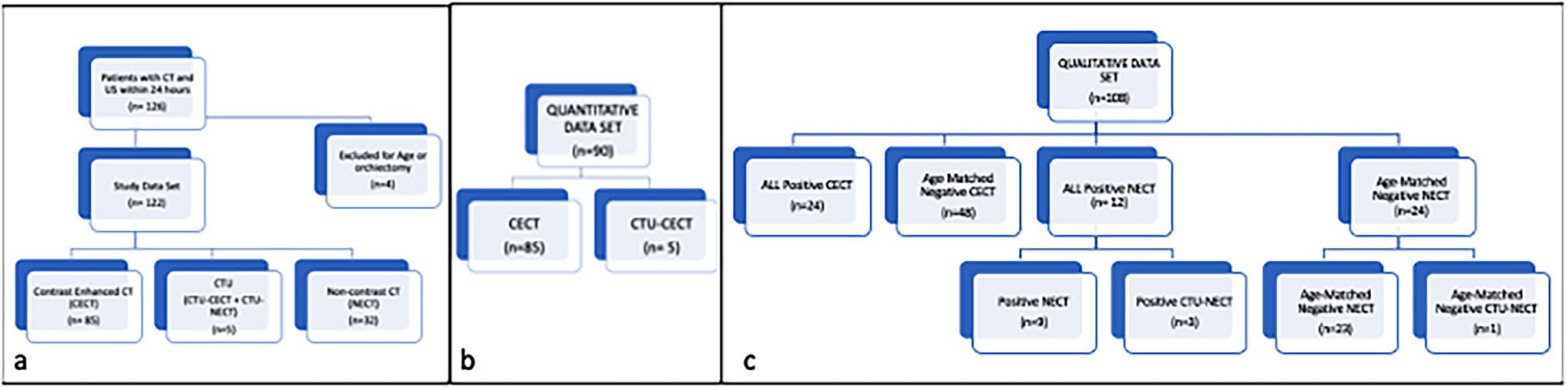
Study Dataset Flowchart. CECT = contrast enhanced CT; NECT = non-enhanced CT. CTU = CT Urogram consisting of both NECT and CECT datasets. Total dataset (**a**), Quantitative dataset including all positive and negative CECT (**b**), and Qualitative dataset including all positive cases and age-matched subset of negative exams (**c**)

The electronic radiology database was queried to identify male patients who underwent emergent abdominopelvic CT and scrotal ultrasound within a 24-h timeframe in our five-hospital system between January 1, 2017, and December 31, 2018, including a busy regional tertiary referral center. Non-contrast, contrast, and combined non-contrast/contrast CT exams (CTU) were included. A total of 126 patients were identified. Four patients were excluded due to age < 18 (2) or prior orchiectomy (2), with the remaining 122 patients comprising the study dataset. Of these, 85 had CECT, 32 had NECT, and 5 had combined CTU, summarized in Fig. 1a. From this initial patient cohort, all CECT exams (n = 85) and the post-contrast portions of CTU exams (CTU-CECT, n = 5) were included in the quantitative dataset (n = 90), summarized in Fig. 1b. Subsequently, a qualitative dataset was created comprised of all sonographically confirmed cases of acute scrotal pathology (n = 36), including CECT (n = 24), NECT (n = 9), and non-contrast portions of CTU exams (CTU-NECT, n = 3), as well as a randomized, age-matched selection of sonographically negative patients (n = 72), summarized in Fig. 1c. Cases were classified as positive for symptomatic scrotal pathology (SSP) if: (1) there was an ultrasound documenting scrotal pathology performed within 24 h of the CT; and (2) the abnormality was clinically determined to represent the etiology for patient’s symptoms in the electronic medical record (EMR). Scrotal abnormalities discovered at ultrasound, but considered clinically incidental in the EMR, were graded as negative for SSP. Patients without abnormalities at ultrasound were also classified as negative for SSP.

### Imaging technique

CT examinations were performed on a variety of multidetector platforms (Siemens AS 64, Siemens Medical Solutions, Malvern, PA; Toshiba Aquilion One and Toshiba Aquilion Prime; Toshiba/Canon Medical Systems, Tustin, CA) using routine clinical protocols. Axial images were acquired at 2–3 mm slice thickness/interval, with equivalent sagittal and coronal reconstruction parameters. For CECT exams, weight-based contrast dosing was employed with administration of Isovue 370 (Bracco Diagnostics, Princeton, NJ) at an injection rate of 2.5-5 cc/sec using a mechanical power injector (MedRad/Bayer Healthcare, Indianola, PA). Images were acquired with semi-automated, automated, or fixed timing following administration of intravenous contrast depending on protocol (including CT angiography, routine abdominopelvic CT, and CTU protocols).

Ultrasound exams were performed using routine clinical protocols on a variety of ultrasound machines (Acuson Sequoia, Siemens Medical, Malvern PA; and Philips iU2, Philips Healthcare, Andover, MA). All exams were performed by sonographers using high frequency linear arrays and included both gray-scale and color Doppler imaging.

### Quantitative analysis

**Fig. 2:**
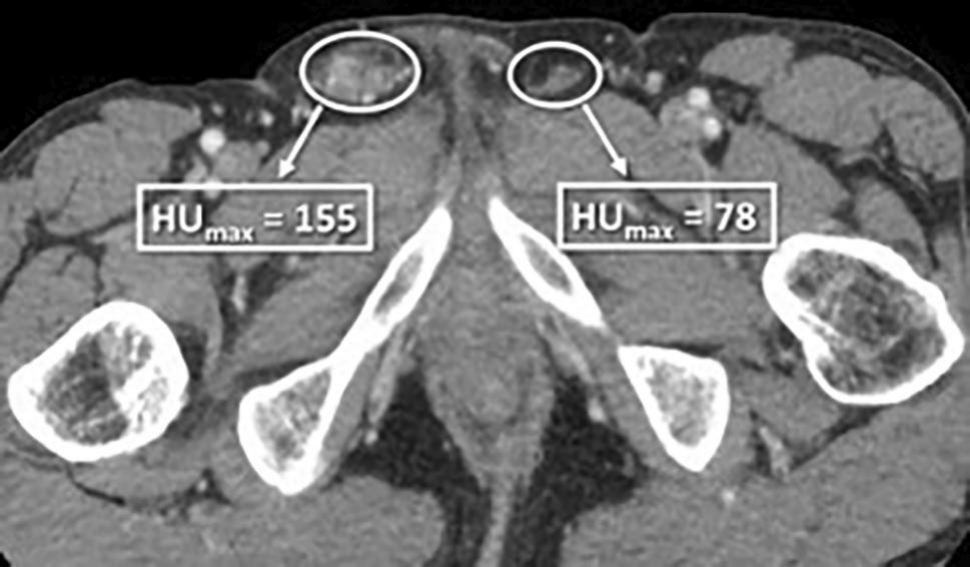
58-year-old with postoperative pelvic and scrotal swelling and pain with one of two contiguous axial CT images best depicting spermatic cord shown with ROI’s encompassing the cords. Differential enhancement was 50% higher on the right in this patient with right-sided epididymitis

One radiologist (RTW) performed all contrast-enhanced CT quantitative analysis (CECT and CTU-CECT exams). For each study, two contiguous axial slices best depicting both right and left spermatic cords were selected. Subsequently, regions of interest (ROI) were manually drawn on the slices encompassing each spermatic cord but excluding extraneous inguinal canal contents (e.g., fluid, bowel, fat), and maximum Hounsfield Unit density (HUmax) recorded (Fig. 2). Ipsilateral HUmax values were averaged, and the mean utilized for statistical analysis.

### Qualitative analysis

Qualitative analysis of each case was performed independently by three fellowship-trained body radiologists blinded to the diagnosis (RTW, JFW, DHS), with 5-, 6-, and 23-years’ experience, respectively. Images for all ultrasound-confirmed positive cases imaged with CECT, NECT and CTU and an age-matched cohort of negative reference cases were reviewed during qualitative analysis. Only the non-contrast component of CTU exams was included for qualitative analysis (to avoid confounding statistical analysis with contrast enhanced CTU components used in quantitative analysis). To achieve statistical power, twice the number of positive cases were selected from the pool of age-matched negative reference studies to serve as controls. Two months following completion of the quantitative analysis, all readers assessed qualitative cases for abnormalities of the spermatic cord. Readers were instructed to grade the spermatic cord as normal or abnormal based on a subjective assessment of cord enlargement and inflammatory change (all studies), or cord hyperenhancement (CECT exams). Sidedness of any cord abnormality was recorded (left, right or bilateral). Reader confidence was reported on a 5-point Likert scale.

### Statistical analysis

Patient ages were compared with an independent samples t-test for true versus positive cases and with an ANOVA with Sidak post hoc comparisons for true versus positive and contrast versus non-contrast cases. Laterality of positive cases was evaluated with binomial tests. Mann–Whitney U tests were used to compare contrast versus non-contrast group differences, as well as reader confidence levels for contrast versus non-contrast images. ROC curves were constructed for the quantitative data and qualitative reader evaluations with area under the curve (AUC) for both maximum cord enhancement and differential enhancement calculated, and diagnostic cutoff points identified. Inter-reader reliability was evaluated with the Intraclass Correlation Coefficient (ICC). Categorical data were evaluated with chi-square tests using a Fisher’s Exact Test for observations with fewer than 5 cases. Two-sided *P* values were reported, and statistical significance was set at the α < 0.05 threshold. Analyses were conducted with SPSS version 27 (IBM: Armonk, NY).

## Results

### Subjects

36 patients were diagnosed with SSP at ultrasound, 83% unilateral, and 58% epididymitis and/or orchitis. Other SSP included mass, hematoma, complex hydrocele, symptomatic varicocele, inflamed inguinal hernia, and soft tissue infection/ Fournier’s gangrene (Table 1).

**Table 1:** Subject demographics, case distribution, and sonographic diagnosis

Variable	Value
Age, all patients (years; mean ± SD)	47.8 ± 15.5
Age, positive cases (years; mean ± SD)	50.1 ± 15.1
Age, negative cases (years; mean ± SD)	46.8 ± 15.6
All patients	122
Patients with CECT	85
Patients with NECT	32
Patients with CTU (combined NECT/CECT)	5
Positive cases, Total	36
Positive cases, CECT	24
Unilateral right	12
Unilateral left	9
Bilateral	3
Positive cases, NECT	9
Unilateral right	5
Unilateral left	1
Bilateral	3
Positive cases, CTU (combined NECT/CECT)	3
Unilateral right	1
Unilateral left	2
Bilateral	0
Negative cases, total	86
Negative cases, CECT	61
Negative cases, NECT	23
Negative cases, CTU (CECT + NECT)	2
All positive cases, unilateral right	18
All positive cases, unilateral left	12
All positive cases, bilateral	6
Epididymitis and/or orchitis	21
Testicular or scrotal mass	6
Other scrotal infection/inflammation^a^	4
Symptomatic varicocele	2
Miscellaneous^b^	3

^a^Complex inflammatory hydrocele (2), scrotal/penile abscess (1), and Fournier gangrene (1)

^b^Spermatic cord hematoma (2) and inflamed bowel-containing inguinal hernia (1)

### Quantitative results

**Fig. 3:**
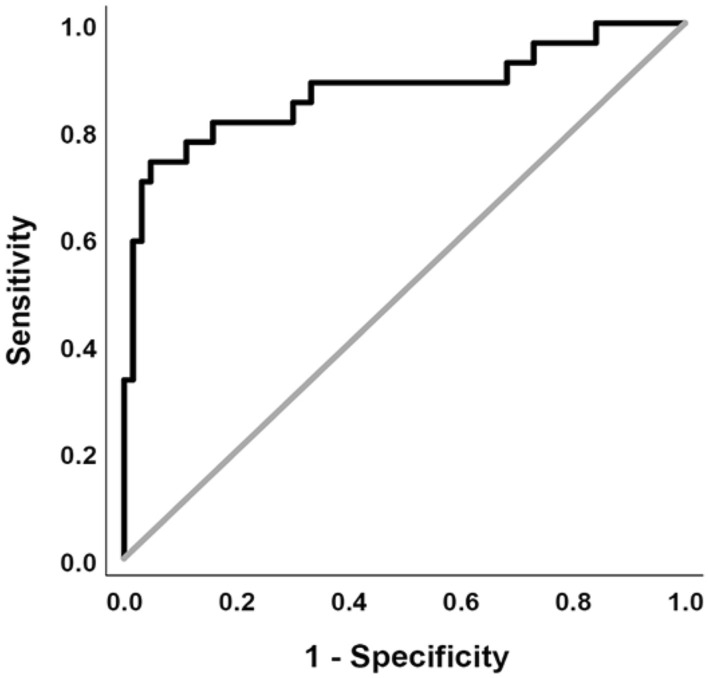
Quantitative ROC curve for spermatic cord differential enhancement, AUC = 0.874, *P* < 0.001

In the quantitative patient cohort, 27% of patients were diagnosed with SSP, with no significant difference in ages between positive and negative subjects (*P* = 0.68). Among positive cases, the etiology was right-sided in 48.1% (13/27), left-sided in 40.7% (11/27), and bilateral in 11.1% (3/27). In patients with unilateral SSP, the positive side was significantly more likely to have a higher peak cord enhancement (HUmax) (23/24; 95.8%) than the negative side (1/24; 4.2%), *P* < 0.001. When the magnitude of enhancement was examined in all positive patients, the positive side (Mdn = 114.50, MAD = 21.00) also showed significantly higher HUmax enhancement than the negative side (Mdn = 77.25, MAD = 8.50), *P* < 0.001. Among positive CECT cases, 59.3% (16/27) were due to infectious or inflammatory etiologies. Neither peak cord enhancement (*P* = 0.85), differential cord enhancement (*P* = 1.00), nor a 25% enhancement threshold (*P* = 1.00) were associated with a specific diagnosis in cases of SSP. A receiver operating curve for differential cord enhancement created from the quantitative dataset was highly discriminatory for SSP, with the resultant area under the curve calculated as 0.874, *P* < 0.001, Fig. 3.

**Fig. 4:**
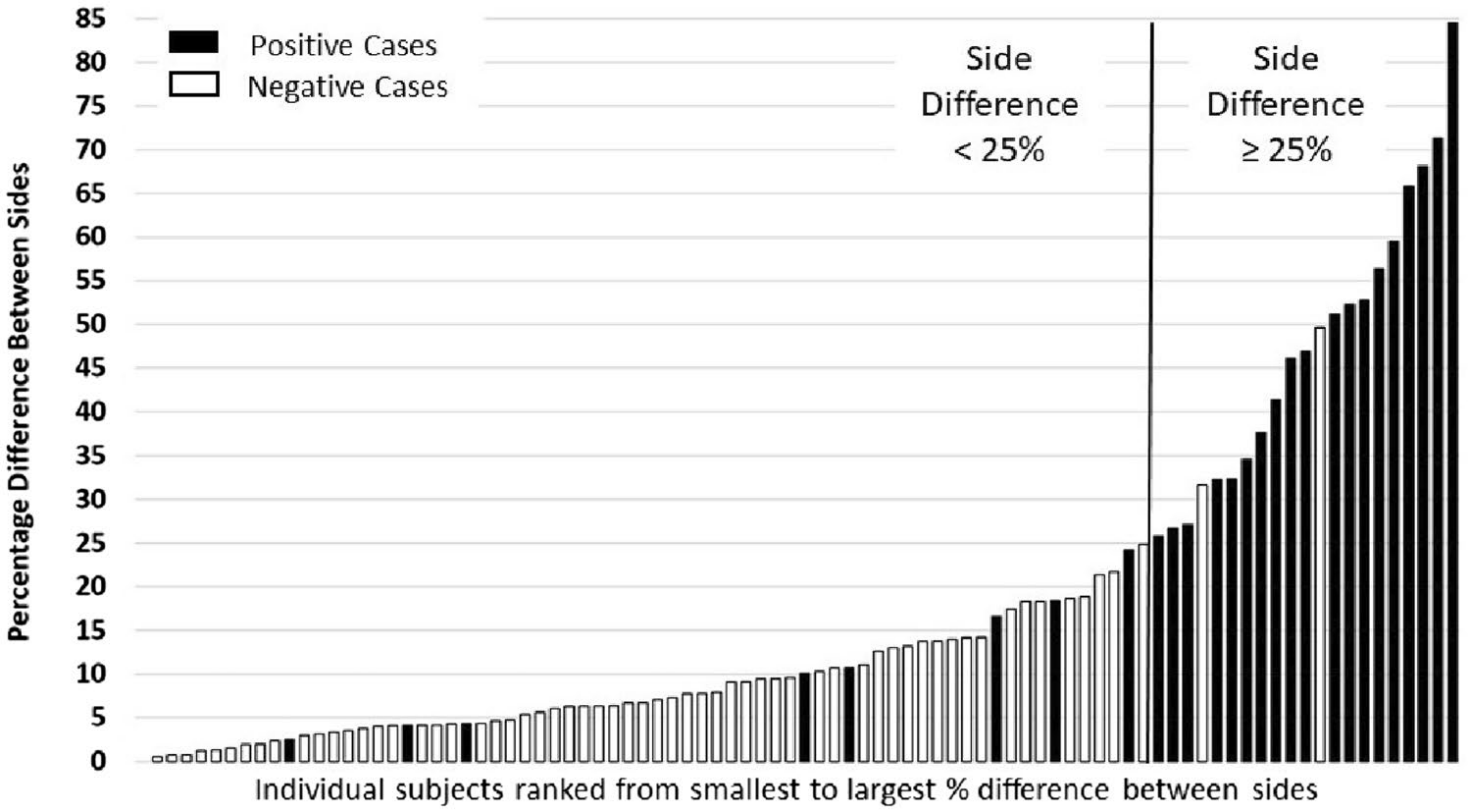
Spermatic cord differential enhancement in patients with (black) and without (white) SSP. Maximum accuracy is illustrated at a 25% enhancement threshold

Using the quantitative AUC curve, cut-off values of various levels of differential enhancement were assessed for relative sensitivity, specificity, positive predictive value (PPV), negative predictive value (NPV), and accuracy for the diagnosis of SSP, Table 2. A 25% differential enhancement threshold yielded the highest diagnostic accuracy (88.9%), excluding negative patients 97% of the time (specificity = 96.8%). Specificity and positive predictive values were maximized with a threshold of 35%, whereas sensitivity was maximized at a threshold of 10%. For the 3 cases of bilateral SSP, none were above the 25% differential enhancement threshold, and only one was above the 10% cutoff (mean: 5.7; range: 2.5 – 10.2). Figure 4 presents the distribution of differential enhancement in positive and negative cases, including an illustration of maximum accuracy at a 25% enhancement threshold.

**Table 2:** Calculated performance of different differential enhancement thresholds for prediction of symptomatic scrotal pathology

% Difference	Sensitivity	Specificity	PPV	NPV	Accuracy
≥ 10	88.9%	66.7%	53.3%	93.3%	73.3%
≥ 15	81.5%	84.1%	68.8%	91.4%	83.3%
≥ 20	74.1%	92.1%	80.0%	89.2%	86.7%
≥ 25	70.4%	96.8%	90.5%	88.4%	88.9%
≥ 30	59.3%	96.8%	88.9%	84.7%	85.6%
≥ 35	48.1%	98.4%	92.9%	81.6%	83.3%
≥ 40	44.4%	98.4%	92.3%	80.5%	82.2%

*PPV* positive predictive value, *NPV* negative predictive value

### Qualitative results

**Fig. 5:**
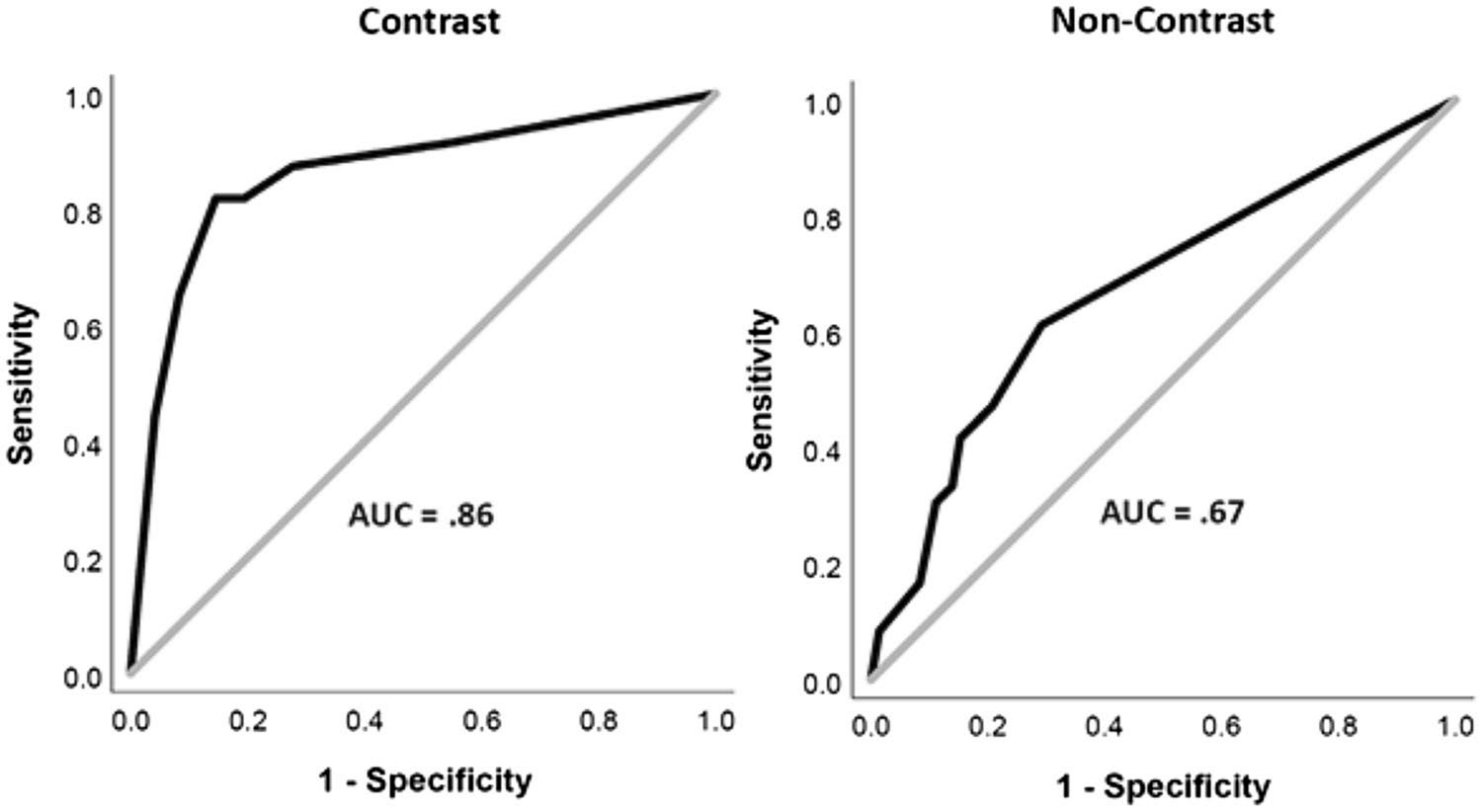
Combined reader ROC curves for CECT and NECT qualitative reads

**Fig. 6:**
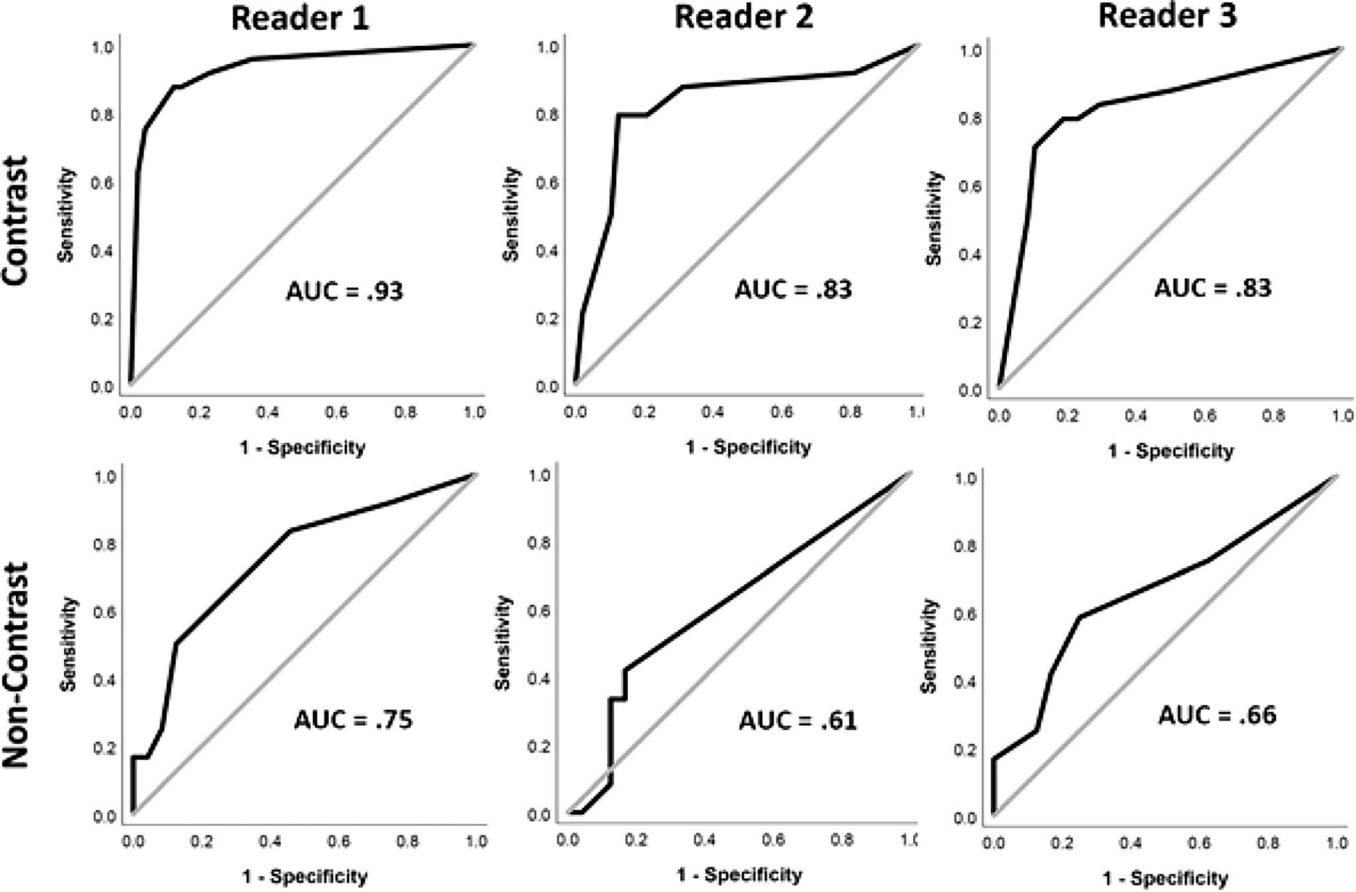
Individual reader ROC curves for CECT and NECT qualitative reads

**Fig. 7:**
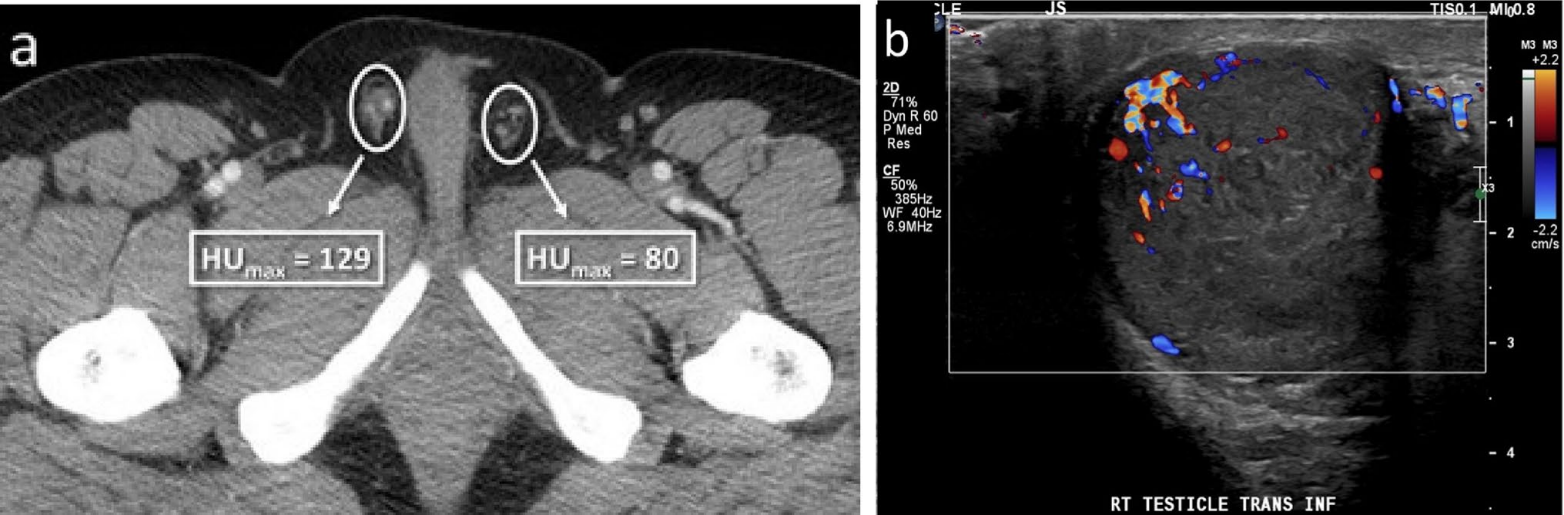
38-year-old patient with vague right-sided abdominal pain and testicular swelling. Quantitative analysis showed 62% greater right-sided differential enhancement (**a**). All readers reported an abnormal right cord with high confidence (5 on a 5-point Likert scale) with asymmetric enhancement and inflammatory change visible at CECT. Color Doppler ultrasound showed right testicular embryonal cell carcinoma (**b**). Cord abnormalities were not appreciated at the prospective CT read

**Fig. 8:**
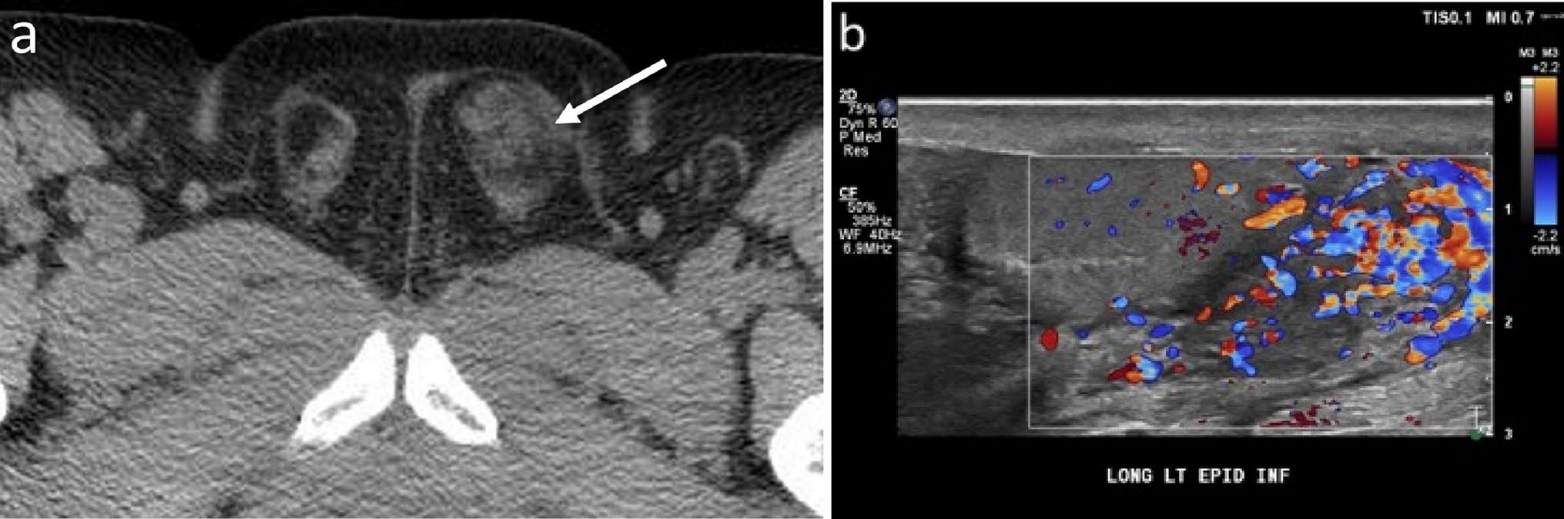
59-year-old patient with hematuria, pyuria and pain radiating into scrotum. All readers reported an abnormal left cord with cord enlargement and inflammatory change visible at NECT (arrow, **a**). Reader confidence levels were lower, ranging from 1 to 3 on a 5-point Likert scale. Color Doppler ultrasound confirmed left-sided epididymitis (**b**). Cord abnormalities were not appreciated at the prospective CT read

**Fig. 9:**
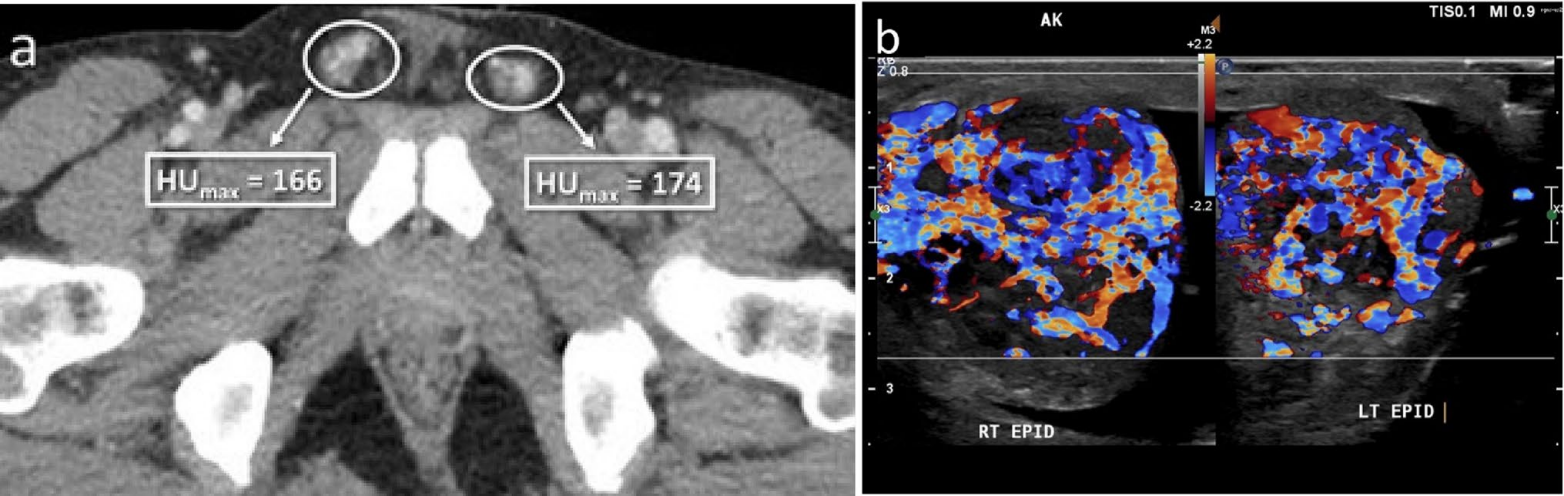
35-year-old patient with scrotal swelling, adenopathy, and elevated ESR. Quantitative analysis showed high bilateral HUmax, but differential enhancement of only 5% (**a**). All readers reported bilateral cord abnormalities with hyperemia and inflammatory change visible at CECT. Reader confidence ranged from 3 to 5 on a 5-point Likert scale. Color Doppler ultrasound confirmed bilateral epididymo-orchitis (**b**). Cord abnormalities were not appreciated at the prospective CT read

In the qualitative patient cohort, 33% of patients were diagnosed with SSP, with no significant difference in ages between positive and negative subjects (CECT p = 0.61, NECT *P* = 0.24). Further, there was no significant difference in ages of CECT and NECT subjects (*P* = 0.24)**.** In the aggregate (Fig. 5) and for each individual reader (Fig. 6), readers had statistically significant AUC values for CECT cases, showing qualitative reads to be highly discriminatory for SSP, with an aggregate AUC of 0.86 and individual reader AUCs ranging from 0.83–0.93. In comparison, while also statistically significant, NECT performance was only poorly discriminatory (aggregate AUC = 0.67; *P* = 0.002). In addition, NECT AUC values were only statistically significant for all readers combined and Reader 1, but not for Reader 2 or Reader 3 (Table 3). Representative CECT and NECT cases are shown in Figs. 7, 8, 9.

**Table 3:** AUC values, 95% Confidence Intervals, and *P* values for qualitative reader ROC curves

	CECT			NECT		
	AUC	95% CI	*P* value	AUC	95% CI	*P* value
Reader 1	0.93	0.86–1.00	< 0.001	0.75	0.57–0.92	0.006
Reader 2	0.83	0.71–0.94	< 0.001	0.61	0.41–0.81	0.30
Reader 3	0.83	0.72–0.94	< 0.001	0.66	0.46–0.86	0.12
All readers	0.86	0.81–0.92	< 0.001	0.67	0.56–0.78	0.002

**Fig.10:**
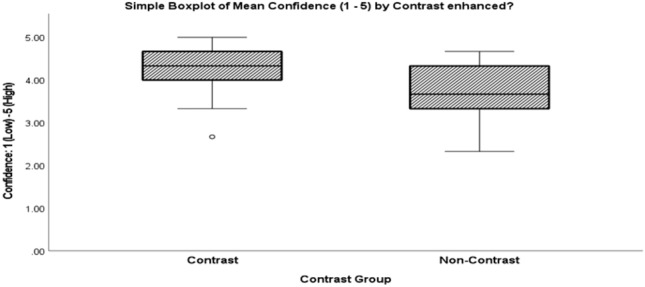
Reader confidence for contrast-enhanced and non-contrast studies including all positive and negative cases, statistically higher at CECT

A high degree of reader agreement for detection of an abnormal spermatic cord was seen for both contrast (ICC = 0.981; 95% CI: 0.972–0.988; F (71, 142) = 53.373, *P* < 0.001) and non-contrast CT (ICC = 0.963, 95% CI: 0.936–0.980; F(35, 70) = 27.076, *P* < 0.001). Figure 10 illustrates overall higher reader confidence with contrast-enhanced CT (Mdn = 4.33, MAD = 0.34) compared to non-contrast imaging (Mdn = 3.67, MAD = 0.66), *P* < 0.001. Table 4 shows the distribution of reader false negatives, with lower specificity at NECT. None of the readers correctly identified 4.2% (3/72) of positive CECT cases compared to 13.9% (5/36) of positive NECT cases. All readers correctly identified 69.4% (50/72) of positive CECT cases compared to 50% (18/36) of NECT cases. Averaged across all three readers, accuracy was significantly higher for CECT (Mdn = 100%, MAD = 0.00) than for NECT (Mdn = 83.34%, MAD = 33.30), *P* = 0.04.

**Table 4:** Qualitative false negative distribution by number of readers

	CECT	NECT
Number of cases correctly identified by 0/3 readers	4.2% (3/72)	13.9% (5/36)
Number of cases correctly identified by 1/3 readers	11.1% (8/72)	11.1% (4/36)
Number of cases correctly identified by 2/3 readers	15.3% (11/72)	25.0% (9/36)
Number of cases correctly identified by 3/3 readers	69.4% (50/72)	50% (18/36)

Bilateral SSP was present in a substantial percentage of cases, including 12.5% (3/24) of CECT and 25% (3/12) of NECT (Table 1). Despite this limitation, readers were able to successfully identify the majority of bilateral abnormalities (Fig. 9). Table 5 shows that bilateral cord abnormalities accounted for between 60 and 62.5% of individual reader’s false negatives at CECT and NECT. The presence or absence of contrast did not impact the distribution of false negative qualitative reads for right, left, or bilaterally positive cases, *P* = 0.32.

**Table 5:** Sidedness of qualitative false negatives for CECT and NECT exams

	CECT				NECT			
	Left	Right	Bilateral	Total	Left	Right	Bilateral	Total
Reader 1	33.3% (1/3)	66.7% (2/3)	0.0% (0/3)	3	50.0% (3/6)	50.0% (3/6)	50.0% (3/6)	6
Reader 2	20.0% (1/5)	20.0% (1/5)	60.0% (3/5)	5	25.0% (2/8)	12.5% (1/8)	62.5% (5/8)	8
Reader 3	20.0% (1/5)	40.0% (2/5)	40.0% (2/5)	5	42.9% (3/7)	14.3% (1/7)	42.9% (3/7)	7
All Readers	23.1% (3/13)	38.5% (5/13)	38.5% (5/13)	13	38.1% (8/21)	14.3% (3/21)	42.9% (10/21)	21

## Discussion

For CECT in the acute care setting, both quantitative and qualitative spermatic cord abnormalities are highly discriminatory for acute scrotal pathology, with AUC values of 0.87 and 0.86, respectively. While we found that NECT can also predict acute scrotal pathology, it is significantly less discriminatory with an AUC value of 0.67. Both CECT and NECT were predictive of a wide range of acute scrotal pathology, most being infectious, inflammatory, or neoplastic. Quantitative differential cord enhancement sub-analysis identified multiple discrete enhancement cutoffs and diagnostic accuracy rates as high as 89%, with the potential to choose thresholds based on a practice’s tolerable miss rate. A high degree of reader agreement was seen for both CECT and NECT (ICC = 0.981 and 0.963, respectively). However, readers reported significantly lower confidence in the diagnosis of cord abnormalities at NECT (4.33 vs 3.67 on a 5-point Likert scale, *P* < 0.001). Quantitative and qualitative performance also suffered in the setting of bilateral SSP, which accounted for up to 63% of readers’ false negatives at qualitative analysis.

While differential spermatic cord enhancement showed robust diagnostic utility in predicting SSP, choice of a diagnostic threshold may depend on the patient population and clinical acuity. In practice, cutoff selection could be tailored to best-address specific clinical priorities. For example, in an outpatient setting with a low pretest probability of SSP, a 35% differential enhancement level maximizing specificity may be preferred to avoid false positives and unnecessary follow-up imaging. Alternatively, in an urgent care setting, there may be a higher likelihood of acute pathology or confounding clinical presentations. In this environment, a differential enhancement level of 10% (emphasizing sensitivity) or 25% (emphasizing accuracy) may be more appropriate. Regardless of threshold value, our quantitative approach has the potential to improve patient care by alerting providers to unsuspected scrotal pathology, leading to more focused treatment, confirmatory imaging, timely urologic consultation, and fewer "bounce-back" care encounters for vague or non-localizing symptoms resulting from SSP.

High performance of any radiologic quantitative measure should also be weighed against reproducibility and ease-of-use. Here, our quantitative analysis was designed for simplicity, using HUmax from only two ROIs encompassing the spermatic cords. Employing HUmax should eliminate the impact of inclusion of fat surrounding the cord (unlike mean HU density measurements), decreasing variability introduced by the size or shape of a manually drawn ROI. Further, HUmax ROI placement may be more rapid than individual cord vessel diameter or intraluminal ROI measurements utilized in prior studies [12, 13]. Additionally, use of differential enhancement analysis may be more reliable than peak cord enhancement in the setting of non-uniform CECT protocols with use of variable iodine concentrations, injection rates and acquisition timing. While assessment of reproducibility and speed of use for quantitative measures is beyond the scope of this study, this could be addressed in a future prospective trial.

Qualitative assessment of the spermatic cord (relying on enlargement, hyperemia, or stranding) had excellent performance, only slightly behind quantitative analysis. While qualitative assessment of NECT was discriminatory for SSP, and its interobserver agreement was high, performance and radiologist confidence were significantly lower than with CECT. This is likely explained by lack of intravascular contrast and its additional diagnostic utility for qualitative assessment of the spermatic cords. As obstructing urolithiasis can present with referred scrotal pain, non-contrast imaging in the setting of SSP is likely common. While the inguinal canals are included on routine CECT and NECT, our findings reinforce the importance of incorporating the inguinal canal in radiologists’ routine search patterns, as both inguinal hernias and scrotal pathology may be prevalent in the acute care setting.

Two prior studies found a similar correlation between CECT spermatic cord abnormalities and unilateral scrotal pathology at ultrasound. In one, scrotal pathology was associated with asymmetric cord vessel size and hyperenhancement, however varicoceles accounted for 60% of positive cases [12]. In the second, scrotal pathology was associated with asymmetric cord enhancement in 62.5% of positive cases (primarily epididymo-orchitis and testicular neoplasm) versus 6.5% of negative cases. In our patient cohort, we also found fewer varicoceles, potentially due to differences in patient selection and the lower incidence of symptomatic varicoceles in the acute-care setting [14]. In that study, mean cord densities were associated with specific scrotal pathologies with differences in enhancement theorized to be related to differential blood supply [13, 15, 16]. We, however, found no significant differences in peak or differential cord enhancement between infectious, inflammatory, and neoplastic etiologies. While Gupta et al. posited that quantitative assessment of cord vessel attenuation and threshold ratios would likely be impractical or impossible at CT, we found differential cord enhancement to be both highly accurate and specific for SSP. Of particular note, neither prior study included cases of bilateral scrotal pathology, which we found in 12.5% of CECT and 25% of NECT positive cases. Unsurprisingly, SSP in these patients was harder to identify using either quantitative or qualitative analysis, likely due to greater symmetry of cord enhancement and morphology. Since bilateral scrotal pathology appears to be relatively common in the acute-care setting, its exclusion could have improved our quantitative and qualitative performance. However, its inclusion in our analysis more accurately reflects patient presentations seen in our clinical practice.

This study had limitations. First, this was a single-center retrospective study with inherent selection bias due to inclusion of only patients with contemporaneous ultrasound and CT. Similar to prior studies, our patient cohort did not include any cases of testicular torsion, likely due to its unambiguous clinical presentation [13]. Presumptively, torsion would show ipsilateral decreased spermatic cord enhancement, but, to our knowledge, this has yet to be reported outside of an experimental model [17]. Our cohort also did not include any cases of funiculitis, which would likely present with ipsilateral spermatic cord hyperemia. Second, there was a relatively small sample size for NECT cases. While our findings of lower NECT accuracy compared to CECT would be expected, a larger number of NECT exams could better confirm our findings. Third, our study CT protocols were heterogeneous, including post-contrast scan timing. While this reflects the day-to-day reality facing many practices, more uniform protocols may have shown better accuracy and/or reader agreement. Finally, while potentially dependent on individual practice parameters, diagnostic performance of defined quantitative thresholds would be better addressed with a future prospective trial.

## Conclusion

Quantitative spermatic cord differential enhancement is a simple technique that accurately predicts SSP, slightly outperforming qualitative analysis at CECT. Differential enhancement thresholds may allow radiologists to prioritize accuracy, sensitivity, or specificity, tailored to individual practice environments. Qualitative findings at NECT can also predict SSP, but with significantly lower accuracy. In the acute-care setting, unilateral and bilateral spermatic cord abnormalities at CT should prompt further clinical assessment and dedicated sonographic evaluation.
